# MEGA-GO: functions prediction of diverse protein sequence length using Multi-scalE Graph Adaptive neural network

**DOI:** 10.1093/bioinformatics/btaf032

**Published:** 2025-01-23

**Authors:** Yujian Lee, Peng Gao, Yongqi Xu, Ziyang Wang, Shuaicheng Li, Jiaxing Chen

**Affiliations:** Guangdong Provincial Key Laboratory IRADS, Beijing Normal University-Hong Kong Baptist University United International College, Zhuhai 519087, China; Department of Computer Science, Beijing Normal University-Hong Kong Baptist University United International College, Zhuhai 519087, China; Department of Computer Science, Beijing Normal University-Hong Kong Baptist University United International College, Zhuhai 519087, China; Department of Computer Science and Technology, Guangdong University of Technology, Guangzhou 510520, China; Department of Science of Chinese Materia Medica, Guangdong Medical University, Dongguan 524023, China; Department of Computer Science, City University of Hong Kong, Hong Kong, China; Guangdong Provincial Key Laboratory IRADS, Beijing Normal University-Hong Kong Baptist University United International College, Zhuhai 519087, China

## Abstract

**Motivation:**

The increasing accessibility of large-scale protein sequences through advanced sequencing technologies has necessitated the development of efficient and accurate methods for predicting protein function. Computational prediction models have emerged as a promising solution to expedite the annotation process. However, despite making significant progress in protein research, graph neural networks face challenges in capturing long-range structural correlations and identifying critical residues in protein graphs. Furthermore, existing models have limitations in effectively predicting the function of newly sequenced proteins that are not included in protein interaction networks. This highlights the need for novel approaches integrating protein structure and sequence data.

**Results:**

We introduce Multi-scalE Graph Adaptive neural network (MEGA-GO), highlighting the capability of capturing diverse protein sequence length features from multiple scales. The unique graph adaptive neural network architecture of MEGA-GO enables a more nuanced extraction of graph structure features, effectively capturing intricate relationships within biological data. Experimental results demonstrate that MEGA-GO outperforms mainstream protein function prediction models in the accuracy of Gene Ontology term classification, yielding 33.4%, 68.9%, and 44.6% of area under the precision-recall curve on biological process, molecular function, and cellular component domains, respectively. The rest of the experimental results reveal that our model consistently surpasses the state-of-the-art methods.

**Availability and implementation:**

The source code and data of MEGA-GO are available at https://github.com/Cheliosoops/MEGA-GO.

## 1 Introduction

Proteins, as products of gene expression, have multifaceted roles in living organisms, acting as catalysts, signal transducers, and structural components (Hopfner and Hornung 2020, Boadu *et al.* 2023). Their implications extend to practical domains like disease management, novel therapeutics, and crop productivity enhancement. However, the scientific community faces an urgent need for rapid and precise protein function annotation tools to analyze vast sequence repositories and uncover latent functionalities (Zhang *et al.* 2024). Only a small fraction of this data has been experimentally annotated (Azli and Mat Isa 2024).

In response to this demand, various computational methods have emerged aimed at automating protein function prediction. In the early stages, machine-learning-based methods such as BLAST and FunFams (Buchfink *et al.* 2015, Das *et al.* 2015) are widely used to transfer functional information between similar sequences, but their predictive capabilities for novel sequences, especially those dissimilar to annotated sequences, are limited. Subsequently, researchers have increasingly turned to deep learning methods, incorporating cutting-edge techniques such as convolutional neural networks (CNNs) (Albawi *et al.* 2017) and graph neural networks (GNNs) (Zhou *et al.* 2020). These innovative approaches effectively integrate diverse information from various sources, including sequences, structures, and domain knowledge, thereby significantly bolstering the accuracy and efficiency of annotation.

Integrating with deep learning models, methods like DeepGo utilizes CNN (Kulmanov *et al.* 2018) has advanced the frontier of protein function prediction by combining features derived from protein sequence matrices and embedding vectors of nodes within protein interaction networks. Moreover, GNN-based methods like DeepFRI, HEAL, and Struct2Go (Gligorijević *et al.* 2021, Gu *et al.* 2023, Jiao *et al.* 2023) reveal that processing protein 3D structure information through GNNs, combined with inferring function and protein contact map predictions, can significantly improve prediction performance. Specifically, DeepFRI utilizes the long short-term memory network (Huang *et al.* 2015) for handling the protein sequence data aiming at modeling complex sequence dependencies and then traversing through GNN for feature extraction. HEAL and Struct2Go are similar when referring to the improvement of GNN feature extraction, with the first one focusing on a structural attention mechanism (Zhou and Li 2021), the second one focusing on a hierarchical pooling technique (Pham *et al.* 2021).

Despite significant advancements in protein function prediction, these GNN-based methods are with limitations. For the input data of GNN, previous work often used models such as node2Vec, SeqVec, ESM-1b protein language model, and one-hot encoding (Chren 1998, Grover and Leskovec 2016, Heinzinger *et al.* 2019, Lin *et al.* 2022) to convert one-dimensional protein sequence data into feature embeddings, followed by a simple fusion operation on these features, which may lead to information redundancy. In terms of the GNN network architecture, one major challenge is the issue of excessive smoothing, leading to nodes becoming similar to each other, preventing the network from capturing the complex details necessary for accurate predictions (Lee *et al.* 2024). Additionally, different sequences of proteins vary in features, even for homologous proteins (Fitch 1970). If the network stacks layers in a sequential manner to extract the features, it will overlook these distinctive features. The presence of these issues exacerbates the overall accuracy of the predictions.

**Figure 1.**
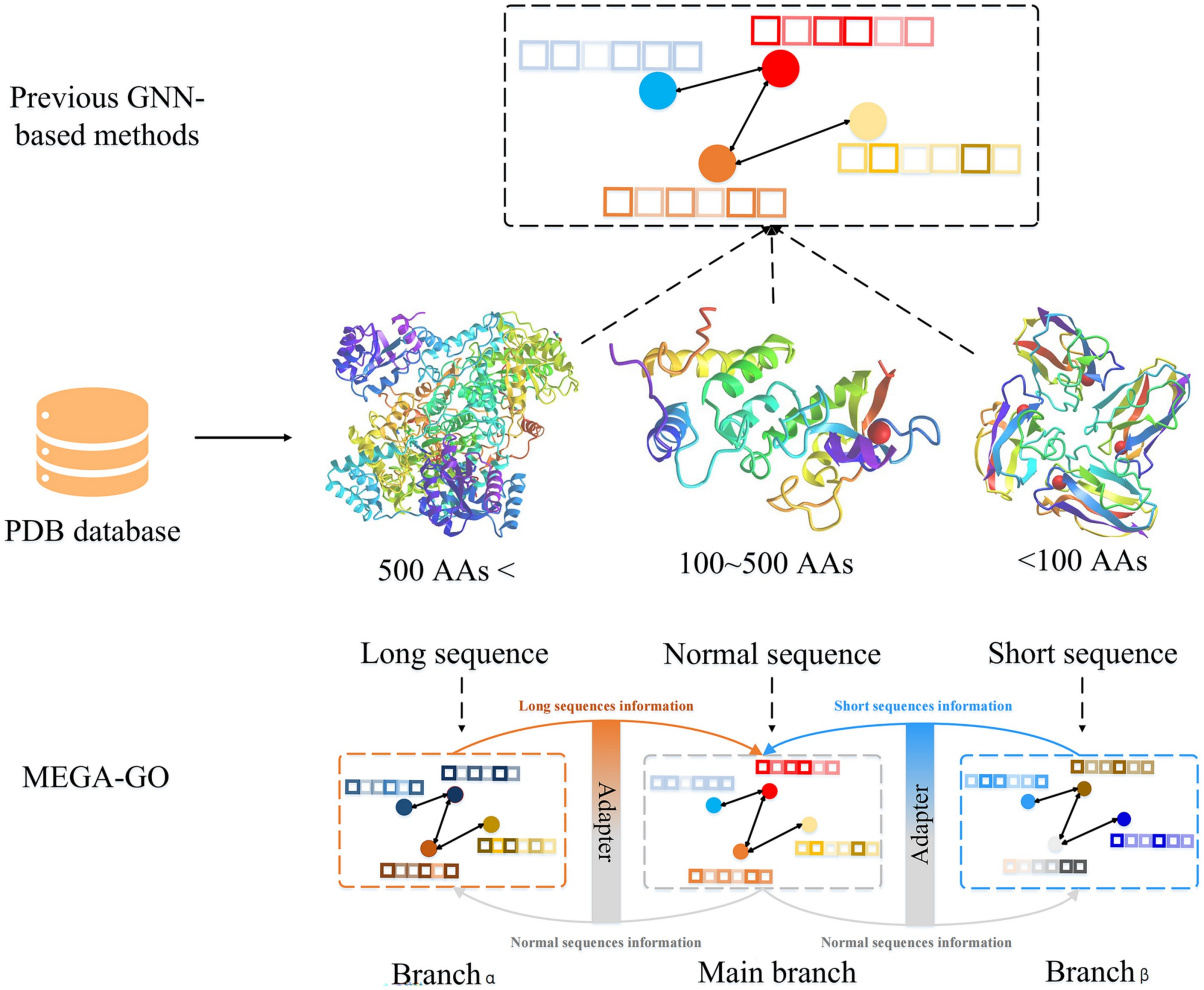
Network architecture comparison of the previous GNN-based methods and MEGA-GO, where the previous work utilizing the GNN-based network for all sequence length of proteins, MEGA-GO have different attention for diverse sequence length of proteins.

To address these limitations, we propose the Multi-scalE Graph Adaptive neural network (MEGA-GO), a novel method for predicting the functions of diverse sequence lengths of protein. Our methodology includes an adaptive feature fusion technique that effectively constructs an informative graph input while reducing information redundancy. Regarding network architecture, Fig. 1 clarifies the differences between the previous GNN-based methods and MEGA-GO. We employ a triple concurrency mechanism for multiscale feature extraction, allowing it to capture the differential features of each protein structure. The Graph SAmple and aggreGatE Network (GraphSAGE) (Hamilton *et al.* 2017) serves as the backbone of the model. The architecture consists of three branches: the Main branch and two additional branches. These branches are designed to pay different attention to proteins with long [more than 500 amino acids (AAs)], normal (100–500 AAs) and short (<100 AAs) (Doolittle 1995) protein sequence lengths. An adapter is incorporated between the Main branch and the two branches, facilitating the integration of different information that each branch focuses on. Additionally, the Main branch’s trunk information is shared with the two branches. This architectural design effectively addresses the over-smoothing problem mentioned earlier and allows for the extraction of protein features from multiple perspectives, enabling the model to learn rich and complex feature information. Furthermore, in the final stage of the network, we introduce an adaptive selection mechanism that prioritizes the most relevant and informative features for feature enhancement, providing comprehensive outputs. Comprehensive experiments including comparative evaluation, sensitive evaluation, and ablation studies have been conducted on real protein data to demonstrate the superiority of MEGA-GO over six state-of-the-art counterparts. The main contributions are three-fold:

- The conversion of raw data to feature embeddings is optimized using an adaptive augmentation and fusion mechanism to generate feature embeddings that capture key information and patterns.
- This is the first attempt to use a concurrent multiscale protein feature extraction mechanism. Each branch in our model uniquely captures and processes proteins of varying sequence lengths.
- We incorporate adapter mechanisms within the network to facilitate information exchange between the branches, enabling knowledge integration that enhances the model’s accuracy and overall performance in protein function prediction tasks.

The rest of this paper is organized as follows: Section 2 provides preliminaries, and Section 3 presents our proposed approach in detail. Experimental results are demonstrated in Section 4 with in-depth analysis. Finally, Section 5 gives the concluding remarks.

## 2 Preliminaries

This section presents necessary pre-definitions of the input graph G, G = (V, E), including node feature extraction and edge inferring in Section 2, and the network architecture using the GNN in Section 2. Table 1 sorts out frequently used symbols in this paper.

**Table 1.** Frequently used symbols.

Symbols	Explanations
G = (V, E)	Input protein graph, containing nodes and an edge list.
V $\in R^{N\times F}$	*N* number of nodes with *F* feature dimensions.
E $\in R^{2\times M}$	Establishment of interactions between nodes.
$h_{v}^{\left(l\right)}$	Output from GraphSAGE, where layer (*l*) = 1, 2, 3.
$h_{v}^{\left(2\right)}(M)$	Output from the Main branch in the second layer.
$h_{v}^{\left(2\right)}(\alpha )$	Output from the ${\text{Extractor}}_{\alpha }$ branch in the second layer.
$h_{v}^{\left(2\right)}(\beta )$	Output from the ${\text{Extractor}}_{\beta }$ branch in the second layer.

### 2.1 Input preparation

Node features (V ∈  $R^{N\times F}$) are extracted through a dual approach that incorporates information from two distinct aspects. For the first aspect, a one-hot residue encoder (Chren 1998) is employed to encode each sequence using amino acid symbols. It ensures a unique representation for each residue, effectively capturing its distinct characteristics. The encoded features are denoted as $V_{\text{One-hot}}$  ∈  $R^{N\times f_{1}}$. The second aspect exploits the ESM-1b protein language model (Lin *et al.* 2022), which is an extensive language model explicitly tailored for proteins, generating intricate residue embeddings, denoted as $V_{\text{ESM}-1b}$  $\in R^{N\times f_{2}}$. Unlike the previous approaches, we merely use two linear layers to fuse *f*_1_ and *f*_2_ to be *F*. Since *F* serves as a potent mechanism to encapsulate the intrinsic knowledge intricately woven into the protein features, we take steps further to adaptively augment and fuse them, as presented in Section 3.

**Figure 2.**
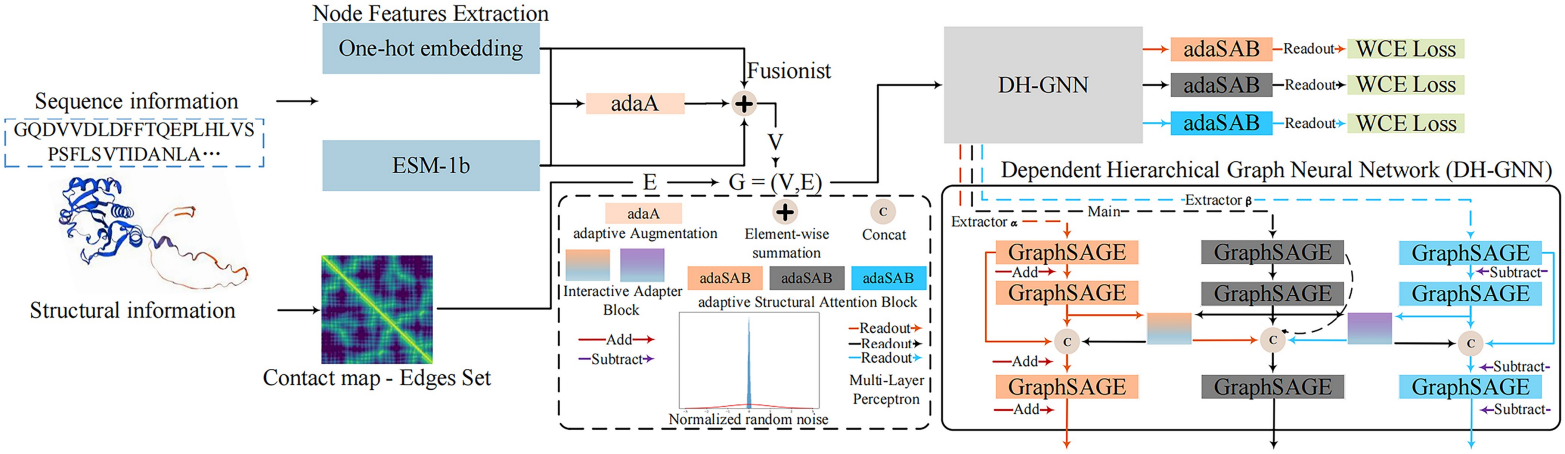
The workflow of MEGA-GO. To acquire the nodes’ features, we have the One-hot encoder and the ESM-1b. Then the adaAF mechanism is proposed to adaptively augment and fuse the protein features, addressing the information redundancy. To generate the Contact map (Edge set E), we use RaptorX. Protein data G is fed to DH-GNN to extract protein features from multiple scales. A normalized random noise signal will be added in ${\text{Extractor}}_{\alpha }$, and subtracted in ${\text{Extractor}}_{\beta }$, resulting in two Extractor branches being more sensitive to long and short-sequence proteins, respectively. Two IABs are built up between the Main branch and the Extractor branches pursuing the goal of information integration and enrichment. After the information is extracted from DH-GNN, we employ the adaSAB, which allows the model to flexibly select the most informative features for enhancement, rather than using a predefined set of features. Eventually, we utilize the Weighted Cross-Entropy Loss (WCE Loss) in each of the branches, allowing for adaptive assignment of importance to labels and samples, coping with diverse protein sequence lengths effectively.

To construct an edge set (E ∈  $R^{2\times M}$, *M* ∈ [0, *N*^2^]) among nodes denoting the “Contact map” in Fig. 2, we initially obtain the 3D atomic coordinates of each protein from Protein Data Bank (PDB) (Berman *et al.* 2000). The subsequent structural network RaptorX (Wang *et al.* 2017) unfolds by establishing edges between nodes based on the spatial proximity of their $C_{\alpha }$ atoms, with an edge added if the distance is <10 Å. This process forms an adjacency matrix ($C_{\alpha }-C_{\alpha }$ contact map), encapsulating the intricate relationships between residues. The matrix can ultimately transform into the edge list E by finding the index of the residues that establish edges.

### 2.2 Network architecture

By compiling protein data to be the input graph G and feeding them into the GNN, the network can guide the information aggregation among nodes by whether they are connected or not. The attributes of G are large data volume and high feature dimension. Of all the GNNs, the Graph SAmple and aggreGatE Network (GraphSAGE) (Hamilton *et al.* 2017) is a memory-efficient and computationally lightweight GNN effective for handling such data (Lo *et al.* 2022). Compared with the method considering the whole graph, it is more scalable. To be more specific, it learns the representation of nodes for the current layer by first randomly sampling several nodes from the neighbor node set *N*(*v*) corresponding to each node *v* in V from the last layer to form *S*(*v*) then applying the aggregation (transformation of a multi-layer perceptron and a mean pooling) to integrate neighboring embeddings, formulating as:

(1) $$h_{S(v)}^{\left(l\right)}=\text{Aggregate}\left(h_{u}^{\left(l-1\right)},\forall u\in S(v)\right).$$

Then concat $h_{S(v)}^{\left(l\right)}$ and $h_{v}^{\left(l-1\right)}$ in

(2) $$h_{v}^{\left(l\right)}=\text{ReLU}\left(W_{\text{sage}}^{\left(l\right)}\cdot \text{Concat}(h_{S(v)}^{\left(l\right)},h_{v}^{\left(l-1\right)})\right)$$

to enrich the node representation ability. Moreover, a learnable weight matrix $W_{\text{sage}}^{\left(l\right)}$ is used to scale the feature to flexibly adapt to the overall graph structure.

## 3 Materials and method

MEGA-GO presented in Fig. 2 first formulates an adaptively augmentation fusionist (adaAF) mechanism in Section 3 to mitigate the information redundancy when integrating *f*_1_ and *f*_2_ into F, and further applies an enhancement. Then introduces the dependent hierarchical message-passing GNN branches (DH-GNN) in Section 3, which composes three H-GNNs, denoted as the Main, ${\text{Extractor}}_{\alpha }$, and ${\text{Extractor}}_{\beta }$, to acquire multiscale of insights within the protein graph G, besides two interactive adapter blocks (IABs) will intervene adapters between the Main branch and the two extractor branches (${\text{Extractor}}_{\alpha },\;{\text{Extractor}}_{\beta }$), allowing for information exchange. Continually, Section 3 presents the adaptive structural attention block (adaSAB), flexibly enhances the feature selection used for enhancement from DH-GNN, and finally the loss function in Section 3.

### 3.1 Adaptive augmentation fusionist

In the previous approaches, *f*_1_ and *f*_2_ are simply using

(3) $$V_{\text{Fuse}}={\text{FC}}_{1}(V_{\text{One-hot}})+{\text{FC}}_{2}(V_{\text{ESM}-1b}),$$

to map the low-dimensional feature (*f*_1_) and the high-dimensional feature (*f*_2_) to the same dimension, then input to the network, leading to information redundancy (Fodor 2002) ($f_{1}\ll f_{2}$), to address this defect, we have the following operations.

To gain individualized features within a batch, we have

(4) $$V_{\text{Fuse}}^{\prime }=V_{\text{Fuse}}-\gamma \cdot \left(\frac{V_{\text{Fuse}}-\mu (V_{\text{Fuse}})}{\sigma (V_{\text{Fuse}})}\right)+\eta .$$

Although normalizing the input variable $V_{\text{Fuse}}$ using its mean *μ*, standard deviation *σ*, scaling factor *γ*, shift factor *η* can reduce the batch effect, to extract insights specific to the normalized distribution, we subtract the normalized output from the original input and enrich the individualized features $V_{\text{Fuse}}^{\prime }$ through two pairs of lightweight augmentation operation.

(5) $$W_{\text{Fuse}}=\text{ReLU}\left({\text{FC}}_{3}(V_{\text{Fuse}}^{\prime })\right)\times 2,$$

returning the attention weight $W_{\text{Fuse}}$ (Zhang *et al.* 2023).

To increase or decrease the significance of the individualized features to$V^{\prime }{}_{\text{Fuse}}$, we have

(6) $$V_{\text{Fuse}}^{\prime \prime }=V_{\text{Fuse}}^{\prime }\cdot W_{\text{Fuse}}.$$

Balancing the contributions of the augmented individualized features and the fused features to the blended representation, we introduce the parameters *δ*_1_ and *δ*_2_ (*δ*_2_ = 1 - *δ*_1_) in Eq. (7). To further optimize the blending, we apply the adaptive average pooling (Pool) technique(McFee *et al.* 2018).

(7) $$V_{\text{adaAF}}=\text{Pool}(\delta_{1}\cdot V_{\text{Fuse}}^{\prime \prime }+\delta_{2}\cdot \text{BN}(V_{\text{Fuse}})).$$

In summary, the issue of redundant information arising from the previous work is effectively addressed by adaAF. By highlighting the overall information, the fused features undergo adaptive selection in the subsequent step while preserving the original features. As a result, adaAF leads to an advanced and refined representation $V_{\text{adaAF}}$ ($V=V_{\text{adaAF}}\in R^{N\times F}$).

### 3.2 Dependent hierarchical GNN

DH-GNN is designed to extract the protein information from multiaspects through the three H-GNN branches (Main, ${\text{Extractor}}_{\alpha }$, and ${\text{Extractor}}_{\beta }$). Branches can cope with protein diversity and solve the specificity of information loss when transmitting through deeper layers in GNN. Besides, by introducing IABs, information can be exchanged adaptively between branches, facilitating mutual enrichment.

#### 3.2.1 Hierarchical GNN

We stack three consecutive GraphSAGE layers (the calculations are presented in Equations (1) and (2) in Section 2) in each of the branches, formulating a hierarchical GNN (H-GNN) in MEGA-GO. Parameter sharing is not performed as a result of extracting $h_{v}^{\left(l\right)}$ from multiple scales minding the protein diversity. To amplify this effect even further, there is a subtle difference in the ${\text{Extractor}}_{\alpha }$, and ${\text{Extractor}}_{\beta }$ branches compared to the Main branch.

A normalized noise is introduced before $h_{v}^{\left(l\right)}(\alpha )$ and $h_{v}^{\left(l\right)}(\beta )$ entering into the next layer, written as:

(8) $$\begin{array}{l} h_{v}^{\left(l\right)}{\left(\alpha \right)}_{\text{noise}+}+=\text{Uniform}\;(\text{noise})\cdot \tau_{1},\\ h_{v}^{\left(l\right)}{\left(\beta \right)}_{\text{noise}-}-=\text{Uniform}\;(\text{noise})\cdot \tau_{2}. \end{array}$$

Such mechanisms are manifested into two key aspects: (i) Adding extra perturbation with the parameter *τ*_1_ and reducing noise with *τ*_2_ can enhance a degree of specificity (Trung *et al.* 2020) and stabilization to the node features, respectively. (ii) The sequence length of different proteins can vary greatly (Tiessen *et al.* 2012), thereby incorporating noise, the model can enhance the feature capture of long-sequence protein data (Zhao *et al.* 2024), while selectively subtracting noise can redirect the model’s attention toward biologically significant features of short-sequence proteins.

Overall, by stacking three GraphSAGEs in branches, H-GNN can expand the receptive field of each node by aggregating the sampled neighbor features. Adding and reducing a certain degree of noise to ${\text{Extractor}}_{\alpha }$ and ${\text{Extractor}}_{\beta }$ can enable our model to focus effectively on protein features of various sequence lengths. And then we will elucidate how to leverage the extracted information to the fullest extent.

#### 3.2.2 Interactive adapter block

When protein data are fed into three H-GNN branches, instead of immediately transmitting information to the third layer, the IAB concluded in Algorithm S1 in Section A is built up between the Main branch and the two extractor branches, to facilitate information blending. Specifically, the Main branch incorporates information from the two extractors, while ${\text{Extractor}}_{\alpha }$ and ${\text{Extractor}}_{\beta }$ coalesce the Main’s information.

We exemplify the information exchange between the Main branch and the ${\text{Extractor}}_{\alpha }$. Specifically, feature representations from the Main branch ($h_{v}^{\left(2\right)}(M)$) with the ${\text{Extractor}}_{\alpha }$ branch ($h_{v}^{\left(2\right)}(\alpha )$), and the Main branch ($h_{v}^{\left(2\right)}(M)$) with the ${\text{Extractor}}_{\beta }$ branch ($h_{v}^{\left(2\right)}(\beta )$) flow in pairs into the IAB. In equation (1) from the Algorithm S1 in Section A, the cosine similarity is utilized to compute the positional weights between pairs. Regarding $h_{v}^{\left(2\right)}(M)$, the similarity vectors ${\text{Sim}}_{\left(M\alpha \right)}$ and ${\text{Sim}}_{\left(M\beta \right)}$ ($\text{Sim}\in R^{N}$) inform the Main branch of the existences of the long and short protein sequence features that warrant greater attention. As for $h_{v}^{\left(2\right)}(\alpha ),\;h_{v}^{\left(2\right)}(\beta )$, the similarity vectors ${\text{Sim}}_{\left(M\alpha \right)}$ and ${\text{Sim}}_{\left(M\beta \right)}$ operate as re-scaling mechanisms, mitigating excessive attention for long, short-sequence lengths of protein features, respectively.

In the remaining steps, the algorithm employs a multiscale feature extraction mechanism through parallel convolutional layers to adaptively capture salient information. The aggregated feature representations are refined and projected into a compact latent space, where attention weights are computed and normalized. These adaptive weights are then integrated into a weighted summation of feature maps, yielding the final adaptive weight matrix (${\text{Weight}}_{\text{adapt}}$). Finally, the outputs ($h_{v}^{\left(2\right)}(M\leftarrow \alpha ),h_{v}^{\left(2\right)}(M\leftarrow \beta ),h_{v}^{\left(2\right)}(\alpha \leftarrow M),h_{v}^{\left(2\right)}(\beta \leftarrow M$) from the two IABs are adaptively enhanced and selected. Furthermore, features enter into the third layer H-GNN are the combination of the previous result:

(9) $$\begin{array}{l} h_{v}^{\left(2\right)}(M)=\text{Cat}\left(h_{v}^{\left(1\right)}(M),h_{v}^{\left(2\right)}(M),h_{v}^{\left(2\right)}(M\leftarrow \alpha ),h_{v}^{\left(2\right)}(M\leftarrow \beta )\right),\\ h_{v}^{\left(2\right)}(\alpha )=\text{Cat}\left(h_{v}^{\left(1\right)}(\alpha ),h_{v}^{\left(2\right)}{\left(\alpha \right)}_{\text{noise}+},h_{v}^{\left(2\right)}(\alpha \leftarrow M)\right),\\ h_{v}^{\left(2\right)}(\beta )=\text{Cat}\left(h_{v}^{\left(1\right)}(\beta ),h_{v}^{\left(2\right)}{\left(\beta \right)}_{\text{noise}-},h_{v}^{\left(2\right)}(\beta \leftarrow M)\right), \end{array}$$

aiming to facilitate the learning of features at various levels of abstraction, resulting in a more comprehensive representation (Guo *et al.* 2019). Consequently, the model can be better equipped to capture the intricate complexities inherent in protein data.

### 3.3 Adaptive structural attention block

SAB has been proven efficient by (Gu *et al.* 2023, Jiao *et al.* 2023). It considers the consecutive attention enhancement to the graph structure G globally and the individual node itself locally. The assigned attention score to each node denotes the structural significance. Characteristics will converge to the node with a higher score, making each protein distinctive. Yet, it cannot deal with proteins of diverse sequence lengths, we made further modifications to the SAB to make each protein more discriminative, denoted as adaSAB, outlined in Algorithm S2 in Section A.

Specifically, in DH-GNN, ${\text{Extractor}}_{\alpha }$ prioritizes long-sequence proteins and preserves original feature information via the presence of IAB. Therefore, we intentionally set the threshold in the formula to be less than *ζ* to ensure such retention regardless of the rest. This principle oppositely applies to ${\text{Extractor}}_{\beta }$. This mechanism maintains a balanced consideration of proteins with different sequence lengths. The attention scores are then normalized to obtain the attention weight. By combining the attention-weighted value vector (attWeight · V) with Q, the model can comprehensively capture the relationship and importance between Q and V. Finally, layer normalization is applied to enhance the model’s stability, and generalization capability (Peng *et al.* 2023).

In conclusion, our goal is to integrate DH-GNN with adaSAB organically. adaSAB serves as a feature integration enhancing mechanism for the diverse features extracted from the Main, ${\text{Extractor}}_{\alpha }$, and ${\text{Extractor}}_{\beta }$ branches. As the two extractor branches have different emphases, we introduce *ζ* to enhance feature selection, ensuring that the ${\text{Extractor}}_{\alpha }$ and ${\text{Extractor}}_{\beta }$ focus on enhancing the most specific features that are relevant to their tasks (long/short-sequence proteins) while still considering the information from the Main branch.

### 3.4 Loss function

In our modeling approach, we employ the weighted binary cross-entropy loss function for multi-label classification tasks (Zhang and Sabuncu 2018), denoted as:

(10) $$\begin{array}{l} \text{WCE}\;\text{Loss}=-\frac{1}{n^{2}}\sum_{i=1}^{n}\sum_{j=1}^{m}w_{j}\cdot (y_{ij}\cdot \;\text{log}({\hat{y}}_{ij})\\ \;+(1-y_{ij})\cdot \;\text{log}(1-{\hat{y}}_{ij})). \end{array}$$

In equation (10), *n* and m denote the number of samples and labels correspondingly. $y_{ij}$ represents the ground truth for label j in sample i, ${\hat{y}}_{ij}$ is the predicted probability of label j in sample i, and $w_{j}$ is the weight for label j written as:

(11) $$w_{j}=\max\left(1,\min\left(10,\sum_{i=1}^{l}\frac{N_{i}^{+}}{l\cdot N_{j}^{+}}\right)\right),$$

where $N_{j}^{+}$ denotes the number of positive instances for label j and l represents the total number of labels. The inclusion of *w_j_* is beneficial in protein functional prediction tasks where proteins have multiple functions. It addresses challenges such as class imbalance, varying functional importance, and asymmetric error costs by assigning higher weights to minority classes, ensuring attention to underrepresented functions (Rezaei-Dastjerdehei *et al.* 2020). The overall loss function L is expressed as:

(12) $$L={\text{WCE}\;\text{Loss}}_{\text{Main}}+{\text{WCE}\;\text{Loss}}_{\alpha }+{\text{WCE}\;\text{Loss}}_{\beta }.$$

## 4 Results

### 4.1 Experimental settings

**Four types of experiments:** (i) comparative study, (ii) concept verification study, (iii) ablation study, and (iv) parameters sensitive study are designed to comprehensively evaluate the efficacy of MEGA-GO.

**Seven counterparts** including three conventional approaches, i.e. BLAST (Buchfink *et al.* 2015), kNN (Cosma 2022), and FunFams (Das *et al.* 2015), and four state-of-the-art approaches, i.e. DeepGO (Kulmanov *et al.* 2018), DeepFRI (Gligorijević *et al.* 2021), HEAL (Gu *et al.* 2023), and Struct2Go (Jiao *et al.* 2023) are chosen for comparison.

**The dataset** comprises 281 416 protein structures obtained from the PDB database (Berman *et al.* 2000) and the SWISS-MODEL repository (Guex and Peitsch 1997) ensuring the input data have multiscale of protein sequence length, denoted as PDBch and SMch, respectively. Moreover, these structures are representative and non-redundant, selected through clustering based on a 95% sequence identity threshold. The dataset includes functional annotations labeled with Gene Ontology (GO) terms [The GO term annotations are retrieved from SIFTS and the UniProtKB (Boutet *et al.* 2016, Dana *et al.* 2019)], specifically 1943 biological process (BP) terms, 489 molecular function (MF) terms, and 320 cellular component (CC) terms. Figure S1 in Section B presents the statistics on the distribution of long, normal, and short protein sequences within the entire dataset. From this, the long and short sequences account for only 9.5% and 8.2% of the total, respectively, leading to the sequence length imbalance ratio. To ensure that MEGA-GO can effectively handle diverse protein sequences, further processing of the dataset is undertaken. Using stratified random sampling, the protein sequence length of the whole dataset is divided by long, normal, and short-sequence labels first, and then each stratum is randomly sampled independently with the same sampling ratio so that the protein sequence ratio of the last three sequence lengths ratio is approximately 1:1:1. To further examine whether recent advancements in protein structure prediction enhance data augmentation, we focused on 44 137 proteins associated with low-frequency GO terms [information content (IC) < 10] in the PDBch training set. The calculation of IC to each GO term (go) is formulated as:

(13) $$\text{IC}(\text{go})=-{\;\text{log}\;}_{2}(P(\text{go})),\;\text{go}\in \text{GO}.$$

A larger IC value indicates lower frequency in the annotated dataset, signifying higher specificity, and vice versa. We then retrieved their structures predicted by AlphaFold2 (AF2) from the AlphaFold Protein Structure Database (Varadi *et al.* 2022). These protein chains collectively comprise the AFch dataset.

**Three evaluation metrics**, namely Fmax, area under precision and recall curve (AUPR), and Smin are used to measure the performance of the model. Among these, Fmax reflects the highest F1 score, AUPR assesses the precision-recall trade-off, and Smin measures the semantic distance between predicted and true GO annotations. A higher Fmax, AUPR, and lower Smin indicate a superior performance in predicting protein function. Experiments are conducted using a 2.90 GHz Intel i7-10700F CPU and an NVIDIA A100 graphics card.

### 4.2 Comparative study

**Figure 3.**
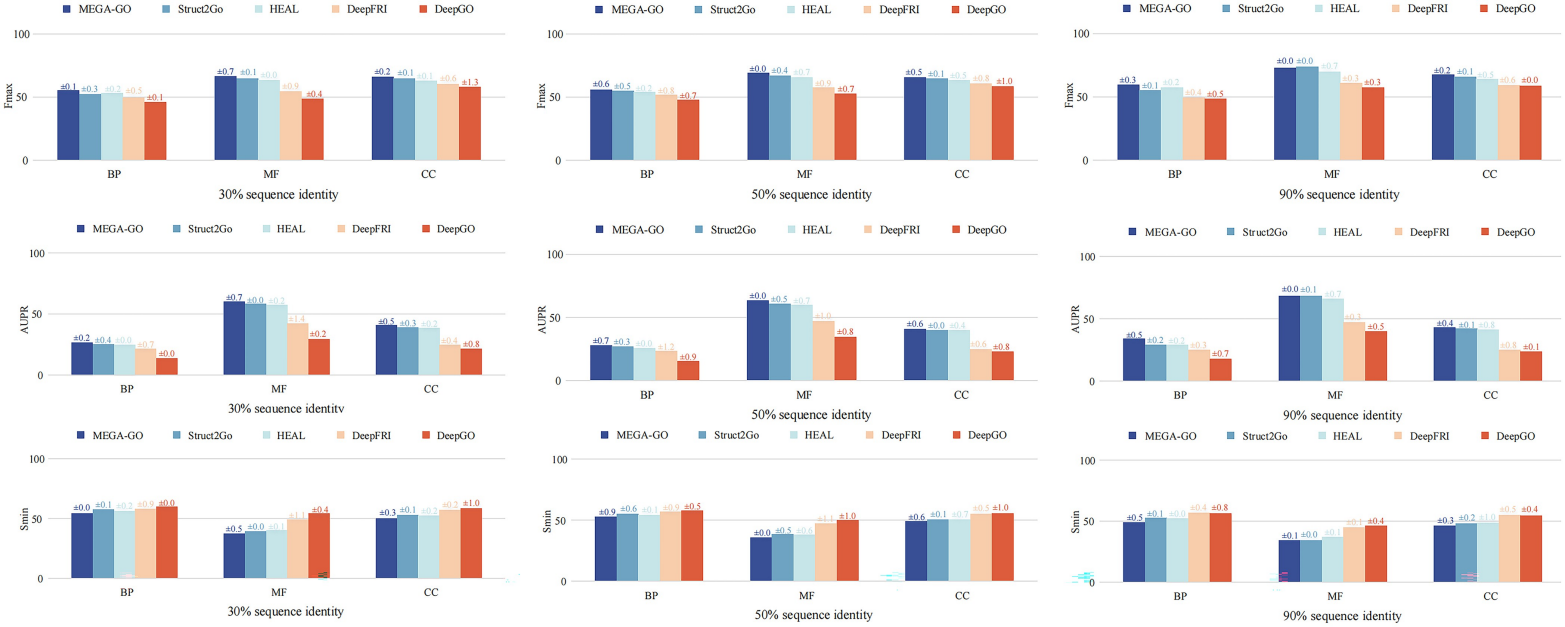
Generalization ability comparison of BP, MF, and CC on Fmax, AUPR, and Smin using 30%, 50%, and 90%, respectively, sequence identity threshold on PDBch test set.

**Figure 4.**
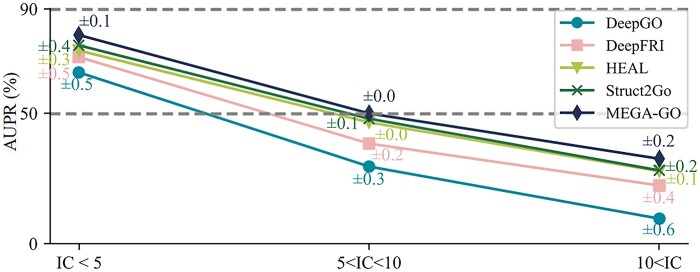
Three IC range sets of BP, MF, and CC terms on models’ AUPR on PDBch test set.

The performance and generalization ability of the models on the PDBch test set, evaluated using Fmax, AUPR, and Smin values for BP, MF, and CC, are presented in Table 2 and Fig. 3. The best and second-best results from the PDBch and SMch datasets are highlighted in bold and underlined, with MEGA-GO PDBch+AFch in Table 2 achieving the highest results among all models utilizing the PDBch and AFch datasets. The dataset is partitioned with training, validation, and testing ratios of 8:1:1 at 95% sequence identity. To assess the models’ generalization ability, the data are further divided into various sequence identity ratios (30%, 50%, and 90%), illustrated in Fig. 3 (results for 40% and 70% ratios can be found in Fig. S2 in Section C). However, even using sequence identity <30% from train to the test set, it does not guarantee that the structures are not nearly identical, we introduce structure identity experiments by utilizing sequence identity of 30% then utilizing the structure identity of 50%, results are shown in Fig. S3 in Section D to compare the 3D structures of proteins. It is possible to more directly understand how similar they are in space, rather than just relying on sequence information, which can further validate the generalization ability of MEGA-GO. Additionally, performance on AlphaFold2 predicted structures are presented in Table S1 in Section E. Furthermore, to evaluate the models’ ability to handle specificity, we present three sets of IC values (Sánchez *et al.* 2011) in Fig. 4. Notably, the results in Figs 3 and 4 are derived from the PDBch and SMch datasets. Since other models also use the PDBch+SMch datasets for accuracy calculations, we similarly use the “MEGA-GO PDBch+SMch” instead of the “MEGA-GO PDBch+AFch” for a fair comparison.

**Table 2.** Model Performance comparison of BP, MF, and CC on Fmax, AUPR, and Smin on PDBch test set.

Model	BP			MF			CC		
	Fmax (↑)	AUPR (↑)	Smin (↓)	Fmax (↑)	AUPR (↑)	Smin (↓)	Fmax (↑)	AUPR (↑)	Smin (↓)
BLAST	31.4 (± 0.9)	5.7 (± 0.3)	63.1 (± 1.1)	30.0 (± 0.6)	14.8 (± 0.4)	64.6 (± 0.1)	43.1 (± 0.2)	9.4 (± 0.4)	65.1 (± 0.3)
kNN	44.2 (± 0.2)	15.6 (± 0.3)	57.2 (± 0.2)	32.5 (± 1.1)	17.3 (± 0.6)	60.0 (± 0.8)	52.2 (± 0.1)	13.9 (± 0.3)	58.5 (± 0.0)
FunFams	50.2 (± 0.6)	25.3 (± 0.4)	56.6 (± 0.7)	57.3 (± 0.2)	38.5 (± 0.5)	52.7 (± 0.5)	61.3 (± 0.9)	26.0 (± 0.2)	56.7 (± 0.5)
DeepGO	51.2 (± 0.5)	20.0 (± 0.9)	54.5 (± 0.2)	58.4 (± 0.7)	41.0 (± 1.0)	45.9 (± 0.7)	59.2 (± 0.6)	24.6 (± 0.7)	53.6 (± 0.7)
DeepFRI	53.6 (± 0.7)	25.4 (± 0.0)	56.0 (± 0.3)	61.3 (± 0.1)	47.2 (± 0.4)	44.4 (± 0.6)	60.0 (± 0.8)	25.6 (± 0.2)	54.0 (± 0.8)
HEAL	57.3 (± 0.6)	29.8 (± 0.3)	51.4 (± 0.7)	72.0 (± 0.3)	66.7 (± 0.0)	36.0 (± 0.2)	64.2 (± 0.1)	41.8 (± 0.6)	47.2 (± 0.0)
Struct2Go	56.5 (± 0.5)	30.1 (± 0.0)	51.6 (± 0.1)	74.7 (± 0.2)	68.2 (± 0.2)	**34.1 (± 0.0)**	66.6 (± 0.0)	42.5 (± 0.1)	47.5 (± 0.6)
MEGA-GO PDBch	55.4 (± 0.3)	29.2(± 0.4)	52.5 (± 0.2)	73.6 (± 0.9)	67.2 (± 1.2)	35.2 (± 0.9)	66.0 (± 0.0)	41.5 (± 0.0)	47.2 (± 0.1)
MEGA-GO PDBch+SMch	**60.3 (± 0.0)**	**33.4 (± 0.4)**	**49.7 (± 0.2)**	**75.8 (± 0.3)**	**68.9 (± 0.4)**	34.8 (± 0.4)	**67.9 (± 0.3)**	**44.6 (± 0.2)**	**46.0 (± 0.1)**
HEAL PDBch+AFch	59.2 (± 0.3)	34.1 (± 0.5)	48.7 (± 0.6)	74.4 (± 0.0)	68.9 (± 0.6)	35.3 (± 0.1)	68.8 (± 0.1)	46.7 (± 0.0)	45.6 (± 0.3)
Struct2Go PDBch+AFch	60.0 (± 0.7)	**34.7 (± 0.6)**	49.2 (± 0.7)	75.8 (± 0.1)	69.0 (± 0.0)	33.5 (± 0.0)	69.3 (± 0.9)	**46.9 (± 0.5)**	42.6 (± 0.7)
MEGA-GO PDBch+AFch	**61.9 (± 0.2)**	34.5 (± 0.1)	**47.0 (± 0.2)**	**77.2 (± 0.5)**	**70.1 (± 0.3)**	**32.2 (± 0.6)**	**70.2 (± 0.1)**	46.8 (± 0.0)	**41.4 (± 0.0)**

The compared models utilize the PDBch+SMch dataset. The version of MEGA-GO PDBch and MEGA-GO PDBch+SMch refers to the PDBch dataset and the PDBch+SMch dataset. PDBch+AFch refers to the utilization of the PDBch and AFch datasets. The best results are highlighted in bold and the second-best results are underlined (% are omitted within the results).

Table 2 provides a comparison between machine learning-based methods (BLAST, kNN, and FunFams) and deep learning-based methods (DeepGO, DeepFRI, HEAL, Struct2Go, and MEGA-GO). The deep models, characterized by a significant number of adjustable parameters, are capable of end-to-end learning and inference from large-scale data. As a result, they generally outperform the machine learning methods in terms of performance. Yet, the choice of the neural network plays a significant role. DeepGO performed worse than the other three because it used 1D convolution, ignoring the long-range dependencies and global information, leading to the model lacking the knowledge of some complex structures of the protein data. The utilization of the GNN can make up for this deficiency; nonetheless, DeepFRI and HEAL models face limitations due to the substantial variation in protein sequence lengths, as relying solely on GNN (GCN specifically) proves inadequate for targeted feature extraction across such diverse sequence lengths. MEGA-GO is therefore designed to address this challenge, where the three branches exhibit varying degrees of attention toward protein sequences of different lengths from equation (8), without excessive focus on any particular length from IAB. Consequently, MEGA-GO reaches the Fmax of 60.3%, 75.8%, 67.9%, AUPR of 33.4%, 68.9%, 44.6%, and Smin of 49.7%, 34.8%, 46.0% on BP, MF, and CC annotation tasks, outperforming the state-of-the-art methods.

The verification of the generalization ability of the models in Fig. 3, BLAST, and FunFams do not involve an explicit division of the training and test sets, therefore, they are not included. Once again, MEGA-GO’s scores consistently suppress the existing methods and notably, the decidable rate of the MEGA-GO is lower than that of either model when sequence identity decreases, demonstrating that the MEGA-GO could model the data effectively even in the case of limited data. The comparative study in Table 2 and Fig. 3 utilizes the PDBch test set, which includes classical and extensively studied proteins. In contrast, the AFch test set encompasses models that have achieved significant breakthroughs in the field of protein structure prediction in recent years. Utilizing this test set allows us to evaluate our model’s performance on contemporary and potentially challenging protein structures. As illustrated in Table S1 in Section E, our model surpasses existing accuracy benchmarks in BP and MF, although the performance of Strcut2Go in CC is slightly lower. These results indicate that MEGA-GO is well-suited for more realistic application scenarios, demonstrating its potential to enhance practical outcomes.

The last proof of MEGA-GO’s performance is the sensitive study to the specificity of IC of the GO term shown in Fig. 4. The PDBch test set is split into three groups based on three range sets of IC of each term belonging to BP, MF, and CC tasks. Results indicate that MEGA-GO achieves a higher AUPR compared to other models for both broader GO terms (IC < 5) and rarer GO terms (10<IC), attributing the perturbation in the ${\text{Extractor}}_{\alpha }$, and ${\text{Extractor}}_{\beta }$ branches using equation (8), which preserves protein sequence features without compromising specificity when message pass through GNN.

### 4.3 Concept verification study

To mitigate the bias arising by random sampling (Jones 2019) from long, short, normal sequences of protein data approximately 1:1:1, we further conduct a cross-validation experiment to obtain the final performance metric of MEGA-GO, presented in Table S2 in Section F. Furthermore, to assess the impact of varying emphases on different protein sequence lengths across distinct branches, we have Table 3. In Table 3, within each dataset group (BP, MF, CC), the best result is in bold, while the second-best is underlined. In horizontal comparison, the top-performing model is highlighted in blue, followed by the second-best in orange. In this experiment, we controlled for variables, specifically, the types of sequence lengths [long, short, normal, and all type (the results of the “All Type” are the same in Table 2)] and the three branches within MEGA-GO [${\text{Extractor}}_{\alpha }$ (for long-sequence protein), Main (for normal sequence protein), and ${\text{Extractor}}_{\beta }$ (for short-sequence protein)], where “MEGA-GO $\alpha_{\times 2}$+M” includes branches ${\text{Extractor}}_{\alpha }$ and Main, “MEGA-GO $\beta_{\times 2}$+M” consists of branches ${\text{Extractor}}_{\beta }$ and Main, and “MEGA-GO $M_{\times 3}$” solely utilizes the Main branch. The term “MEGA-GO” refers to the simultaneous presence of all three branches.

**Table 3.** Model Performance comparison with diverse sequence length of protein data across BP, MF, and CC on AUPR on PDBch test set.

Metric (sequence)	AUPR (long only)			AUPR (short only)			AUPR (normal only)			AUPR (all type)		
	BP	MF	CC	BP	MF	CC	BP	MF	CC	BP	MF	CC
DeepGO	18.2 (± 0.2)	38.7 (± 0.7)	22.6 (± 0.3)	17.0 (± 0.5)	34.9 (± 0.6)	22.9 (± 0.2)	21.2 (± 0.2)_a_	40.5 (± 1.2)_b_	23.1 (± 0.4)_b_	20.0 (± 0.9)_b_	41.0 (± 1.0)_a_	24.6 (± 0.7)_a_
DeepFRI	23.2 (± 0.5)	43.0 (± 0.2)	21.0 (± 0.9)	22.6 (± 0.1)	45.1 (± 0.1)	19.8 (± 0.6)	23.6 (± 0.6)_b_	47.9 (± 0.0)_a_	24.2 (± 0.0)_b_	25.4 (± 0.0)_a_	47.2 (± 0.4)_b_	25.6 (± 0.2)_a_
HEAL	28.8 (± 1.0)	65.2 (± 0.1)	37.8 (± 0.0)	27.1 (± 0.0)	66.0 (± 0.5)	35.2 (± 0.2)	29.0 (± 0.3)_b_	66.5 (± 0.1)_b_	39.7 (± 0.5)_b_	29.8 (± 0.3)_a_	66.7 (± 0.0)_a_	41.8 (± 0.6)_a_
Struct2Go	29.2 (± 0.6)	66.5 (± 0.8)	39.2 (± 0.2)	29.0 (± 0.2)	64.2 (± 1.4)	41.6 (± 0.4)	29.3 (± 0.7)_b_	66.9 (± 0.6)_b_	42.1 (± 0.2)_b_	30.1 (± 0.0)_a_	68.2 (± 0.2)_a_	42.5 (± 0.1)_a_
MEGA-GO $\alpha_{\times 2}$+M	**35.2 (± 0.2)_a_**	**68.9 (± 0.4)_a_**	**45.3 (± 0.8)_a_**	30.8 (± 0.7)	65.9 (± 0.5)	42.2 (± 0.0)	31.0 (± 0.6)	66.5 (± 0.0)	42.8 (± 0.5)	32.4 (± 0.2)_b_	67.3 (± 0.9)_b_	43.1 (± 0.5)_b_
MEGA-GO $\beta_{\times 2}$+M	31.0 (± 0.0)	65.1 (± 0.2)	42.8 (± 1.4)	**35.6 (± 0.7)_a_**	**69.4 (± 1.0)_a_**	**45.0 (± 0.0)_a_**	32.6 (± 0.4)_b_	66.7 (± 0.2)	43.1 (± 0.5)_b_	32.3 (± 0.0)	68.1 (± 0.3)_b_	42.9 (± 0.2)
MEGA-GO $M_{\times 3}$	31.4 (± 0.0)	65.5 (± 0.7)	42.1 (± 0.2)	32.0 (± 0.6)_b_	63.2 (± 0.2)	43.0 (± 0.1)	31.7 (± 1.5)	67.5 (± 0.2)_b_	43.6 (± 0.0)_a_	32.7 (± 0.2)_a_	67.9 (± 0.0)_a_	43.5 (± 0.0)_b_
MEGA-GO	32.5 (± 0.3)	67.3 (± 0.3)	43.8 (± 0.0)	32.3 (± 0.9)	66.0 (± 0.4)	43.0 (± 0.5)	**33.0 (± 0.0)_b_**	**68.0 (± 0.1)_b_**	**44.2 (± 0.5)_b_**	**33.4 (± 0.4)_a_**	**68.9 (± 0.4)_a_**	**44.6 (± 0.2)_a_**

Version of MEGA-GO $\alpha_{\times 2}$+M, $\beta_{\times 2}$+M, and $M_{\times 3}$ stands for the different utilization of the branches, where “$\alpha_{\times 2}$+M” refers to ${\text{Extractor}}_{\alpha }$ and the Main branch, “$\beta_{\times 2}$+M” refers to ${\text{Extractor}}_{\beta }$ and the Main branch, and “$M_{\times 3}$” refers to the Main branch. The best result for each model within one column is highlighted in bold and the second-best result is underlined. The best result within one model on one row is written in X_a_ and the second-best result is written in X_b_ (% is omitted in the result).

The results presented in Table 3 indicate that “MEGA-GO $\alpha_{\times 2}$+M” and “MEGA-GO $\beta_{\times 2}$+M” exhibit superior performance under long and short-sequence types, respectively, underscoring the effectiveness of our design. However, incorporating all types of input into the model, the presence of an imbalance ratio leads to a 2% decrease in the AUPR rate. Furthermore, when comparing these models with their counterparts, it becomes evident that they demonstrate resilience to variations in sequence length, showing that they are not significantly affected by either long or short sequences. This robustness highlights the models’ ability to maintain performance across diverse input conditions, reinforcing the reliability of our approach. The above two studies are conducted under the same conditions as the last subsection.

### 4.4 Ablation study

This subsection contains two parts: (i) to examine the model’s components efficacy, we have three ablated versions of MEGA-GO and (ii) the generalization ability, where the training set is the annotated proteins in 2019, and the test set proteins annotation are ranging from 2019 to 2021. Experiments are conducted under the sequence identity of 95% with the train-validation-test ratio of 8:1:1.

In (i), the three ablated versions of MEGA-GO (H-GNN, ${H-\text{GNN}}_{\times 3}$, and DH-GNN) are compared to verify the effectiveness of DH-GNN. In H-GNN, only the Main branch is utilized. In ${H-\text{GNN}}_{\times 3}$, we utilize three independent branches with the normalized random noise added and subtracted to the ${\text{Extractor}}_{\alpha }$ and ${\text{Extractor}}_{\beta }$, respectively in equation (8), and DH-GNN employs the IAB to generate dependent branches. Additionally, without the usage of adaAF, features are fused by equation (3), and without the utilization of multiple branches, we substitute adaSAB with SAB, excluding the use of *ζ* in Algorithm S2 in Section A. It can be observed from Table 4 that the scores of AUPR stand the highest when utilizing DH-GNN, followed by ${H-\text{GNN}}_{\times 3}$, suggesting that the inclusion of perturbations in ${H-\text{GNN}}_{\times 3}$ lead to enhanced information characteristics in proteins with different sequence lengths extracted from branches and further highlight the importance of integrating information through IAB to achieve comprehensive extraction. Additionally, the incorporation of adaAF and adaSAB in the pre-feature and post-feature extraction stage enhances the AUPR score, thereby underscoring their efficacy and utility.

**Table 4.** Ablation study of MEGA-GO’s components and its corresponding generalization ability on annotated proteins within each year ranking AUPR on BP, MF, and CC.

Settings (proteins annotated in 2019)					AUPR (2019)			AUPR (2020)			AUPR (2021)		
adaAF	H-GNN	${H-\text{GNN}}_{\times 3}$	DH-GNN	adaSAB	BP	MF	CC	BP	MF	CC	BP	MF	CC
	*✓*				17.1	47.4	28.2	12.6	40.9	20.1	9.2	39.3	17.6
*✓*	*✓*				18.8	51.0	33.1	14.6	46.3	29.5	14.2	43.4	26.2
		*✓*			19.5	51.3	31.0	16.3	48.2	28.5	14.8	48.7	26.3
*✓*		*✓*			20.1	53.6	33.7	15.2	50.0	30.4	15.3	52.0	27.6
		*✓*		*✓*	21.3	57.0	35.1	19.7	54.2	32.9	18.2	52.6	27.0
*✓*		*✓*		*✓*	23.4	58.2	38.0	21.5	56.7	34.5	21.0	55.4	30.7
			*✓*		24.2	59.6	34.3	22.5	56.8	36.1	19.8	57.0	30.3
*✓*			*✓*		28.8	63.2	38.0	26.1	59.2	37.6	25.7	59.2	34.3
			*✓*	*✓*	31.6	66.7	41.2	**29.6**	62.1	40.7	26.9	58.8	36.1
*✓*			*✓*	*✓*	**33.4**	**68.9**	**44.6**	29.5	**65.7**	**42.0**	**29.0**	**61.2**	**38.6**

The best result on AUPR within each specific year is highlighted in bold (% is omitted in the result).

In (ii), we aim to evaluate the generalization capability of our proposed method. Consequently, our training data exclusively consists of these 2019-annotated proteins. While the test dataset encompasses proteins annotated from 2019 to 2021. This design aligns with the objectives of the CAFA challenge (Kahanda *et al.* 2015), allowing us to rigorously assess the model’s adaptability to evolving protein annotation landscapes. Through this approach, we seek to determine the robustness and predictive performance of our method against contemporary biological data. Due to the training set comprising annotated proteins from 2019, while the test set includes annotations from 2019 to 2021, there are inherent discrepancies in the protein annotation content across different years. Consequently, the model’s accuracy may experience a certain degree of decline. Yet, MEGA-GO demonstrates commendable performance in handling previously unseen annotated proteins, maintaining a robust AUPR, with declines of 3.8%, 3.2%, and 2.6% on BP, MF, and CC, respectively, when using 2020 annotated protein data as test set; 4.4%, 7.7%, and 6% in 2021. To examine the efficacy of MEGA-GO, we have included an additional temporal hold-out evaluation compared with two existing SOTA methods: HEAL and Struct2Go, presented in Table S3 in Section G.

To further elucidate the impact of the adaAF process described in Section 3 on the input data, as well as the consequential introduction (reduction) of noise to the branches, we have conducted thorough parametric sensitivity analyses.

### 4.5 Parameters sensitive study

MEGA-GO encounters four parameters (*δ*_1_, *δ*_2_, *τ*_1_, *τ*_2_). Parameters *δ*_1_, *δ*_2_ (*δ*_2_ = 1 − *δ*_1_) from equation (7) are the ratio to scale the contribution of the augmented individualized features and the fused features to the overall input. *τ*_1_, and *τ*_2_ are noise proportions of adding and removing to the features in equation (8). These four parameters are used to find the combination to achieve the best result of MEGA-GO, where *δ*_1_  ∈ [10%, 30%, 50%, 70%, 90%] and *τ*_1_, *τ*_2_  ∈ [0.3, 0.5, 0.7, 0.9, 1.5, 2.0].

From Fig. S4a in Section H, by comparing the AUPR scores between excluding and including adaAF, we can conclude that such an approach is effective in better fusing $V_{\text{One-hot}}$ and $V_{\text{ESM}-1b}$, thereby enhancing the model’s processing capabilities. Subsequently, the optimal results are typically observed within the 50%:50% to 70%:30% ratio range, indicating that the input data should encompass both enhanced feature representations as well as the original feature information, to preclude excessive degradation of the inherent IC within the primary feature set. Experiments of results in Fig. S4b in Section H are conducted after the best AUPR result is found in Fig. S4a in Section H. Taking the BP task as an example, the AUPR scores are similar when *τ*_1_ and *τ*_2_ in [0.3, 0.5, 0.7]. In essence, the disparity in the model’s attention to long versus short protein sequences upon the introduction of noise can be attributed to the inherent differences in their feature complexity. Long-sequence proteins encompass a more intricate array of characteristics and short-sequence proteins exhibit relatively simpler features (Romero *et al.* 2001). Introducing noise obfuscates the details of short sequences, forcing the model to focus on and learn long-term global patterns while removing noise preserves short-term local correlations in the protein sequence. However, if overly aggressive perturbations are introduced or reduced, leading to the inherent characteristics being changed, performance will suffer severe degradation.

## 5 Conclusion

This paper presents MEGA-GO, an adapter-based GNN architecture designed to address the challenge of annotating protein functions across varying sequence lengths. MEGA-GO uniquely enhances feature extraction and preserves specificity during message passing, making it the first to handle diverse sequence lengths in protein data. Its superiority over alternative methods is validated by empirical results, with the adaptive adapter modules enabling dynamic feature adjustment to accommodate the heterogeneity in protein samples.

## Supplementary Material

btaf032_Supplementary_Data
