# Supplementary material for: MEGA-GO: functions prediction of diverse protein sequence length using Multi-scalE Graph Adaptive neural network
Source: Bioinformatics. 2025 Jan 23;41(2):btaf032. doi: 10.1093/bioinformatics/btaf032 (PMC11810639; doi:10.1093/bioinformatics/btaf032)
Supplement: btaf032_Supplementary_Data [file btaf032_supplementary_data.zip › 869f3_Supplementary.pdf]

Supplementary

A

Interactive Adapter Block - Algorithm

Interactive Adapter Block (IAB) conducts adaptive feature selection, facilitating feature blending among branches, where the Main branch enhances both long and short-sequence protein features, and Extractor_α and Extractor_β address the challenge of effectively focusing on proteins of varying sequence lengths without excessive concentration on one specific length of protein features.

Algorithm 1 Interactive Adapter Block.

Input: Input features: $\mathbf{h}_v^{(2)}(\text{M})$, $\mathbf{h}_v^{(2)}(\alpha)$; Operation: Convolution (Conv); Kernel list: K = [1,3,5]; Group size: G; Latent dimension: L; Normalization: Batch normalization (BN); Activation function: σ, ϕ; Linear transformation: MLP; Storage list: sl = []; Attention weight list: awl = []
Output: $\mathbf{h}_v^{(2)}(\text{M} \leftarrow \alpha)$, $\mathbf{h}_v^{(2)}(\alpha \leftarrow \text{M})$
MLP has three fully connected layers (FC): [FC_1, FC_2, FC_3]

$$\text{Sim}_{(\text{M}\alpha)}[i] = \frac{\mathbf{h}_v^{(2)}(\text{M})[i] \cdot \mathbf{h}_v^{(2)}(\alpha)[i]}{\left\|\mathbf{h}_v^{(2)}(\text{M})[i]\right\| \cdot \left\|\mathbf{h}_v^{(2)}(\alpha)[i]\right\|} \quad (1)$$

Parallel operation for $\mathbf{h}_v^{(2)}(\text{M})$, $\mathbf{h}_v^{(2)}(\alpha)$, using $\mathbf{h}_v^{(2)}$ instead
$\mathbf{h}_v'^{(2)} \leftarrow \mathbf{h}_v^{(2)} \cdot \text{broadcast}(\text{Sim}_{(\text{M}\alpha)})$
for k in K **do**
  $\sigma(\text{BN}(\text{Conv}(\mathbf{h}_v'^{(2)}, \text{padding} = \text{k // 2})))$
  sl.append($\mathbf{h}_{v\text{k}}'^{(2)}$)
end for
$\text{Feature}_{\text{adapt}} \leftarrow \text{Stack(sl)}$
$\text{S} \leftarrow \text{Sum(sl).mean}$
for FC in MLP **do**
  awl.append(FC(S))
end for
$\text{Weight}_{\text{adapt}} = \phi\,(\text{Stack(awl)})$
return Output $= \text{Feature}_{\text{adapt}} \cdot \text{Weight}_{\text{adapt}}$

adaptive Structural Attention Block - Algorithm

adaptive Structural Attention Block (adaSAB) is an attention mechanism, where K and V are obtained by two independent Graph Convolution Networks (GCN) or FC depending on the selected operation mode (global/local), and Q is a learnable seed vector having the same shape as K, V does. To enable a more effective attention mechanism, the dense operation is applied to transform the 2D features into the 3D format. Then, the attention scores can be computed using Eq. (2). Before

Algorithm 2 adaptive Structural Attention Block.

Input: Input features: $\mathbf{h}_v^{(3)}(\text{M})$, $\mathbf{h}_v^{(3)}(\alpha)$, $\mathbf{h}_v^{(3)}(\beta)$; Seed Vector: Q; Operation: Graph Convolution Network (GCN), GCN_k, GCN_v; Linear transformation: FC_k, FC_v FC; Activation function: σ, ϕ; Normalization: Layer normalization (LN); Operation mode: M = [global, local, global]
Output: $\mathbf{h}_v'^{(3)}(\text{M})$, $\mathbf{h}_v'^{(3)}(\alpha)$, $\mathbf{h}_v'^{(3)}(\beta)$
for m in M **do**
  Parallel operation for $\mathbf{h}_v^{(3)}(\text{M})$, $\mathbf{h}_v^{(3)}(\alpha)$, $\mathbf{h}_v^{(3)}(\beta)$, using $\mathbf{h}_v^{(3)}$ instead
  if m == global **then**
    $\text{K} = \text{Dense}\left(\text{GCN}_\text{k}(\mathbf{h}_v^{(3)})\right), \text{V} = \text{Dense}\left(\text{GCN}_\text{v}(\mathbf{h}_v^{(3)})\right)$
  else
    $\text{K} = \text{Dense}\left(\text{FC}_\text{k}(\mathbf{h}_v^{(3)})\right), \text{V} = \text{Dense}\left(\text{FC}_\text{v}(\mathbf{h}_v^{(3)})\right)$
  end if
  $\text{K} = \text{Cat}\,(\text{Split(K,2)}, 0)\,, \text{V} = \text{Cat}\,(\text{Split(V,2)}, 0)$

$$\text{attScore} = \left(\frac{\text{Q} \cdot \text{K}^\text{T}}{\sqrt{d_\text{K}}} \cdot \text{V}\right) \quad (2)$$

  The three attention scores are denoted as attScore(M), attScore(α), attScore(β)
  attScore'(M) = attScore(M)
  $\text{attScore'}(\alpha) = \begin{cases} \text{attScore}(\alpha), \text{attScore}(\alpha) \leq \zeta \\ 0, \text{attScore}(\alpha) > \zeta \end{cases}$
  $\text{attScore'}(\beta) = \begin{cases} \text{attScore}(\beta), \text{attScore}(\beta) \geq \zeta \\ 0, \text{attScore}(\beta) < \zeta \end{cases}$
  Substitute the three new attention scores with attScore'
  $\text{attWeight} = \phi(\text{attScore'})$
  $\text{Att} = [\text{Q} + \text{attWeight} \cdot \text{V}]_{\text{2Dtransformation}}$
  $\mathbf{h}_v'^{(3)}(\text{M}), \mathbf{h}_v'^{(3)}(\alpha), \mathbf{h}_v'^{(3)}(\beta) = \text{LN}\,(\text{Att} + \sigma((\text{FC(Att)})$
end for
return $\mathbf{h}_v'^{(3)}(\text{M}), \mathbf{h}_v'^{(3)}(\alpha), \mathbf{h}_v'^{(3)}(\beta)$

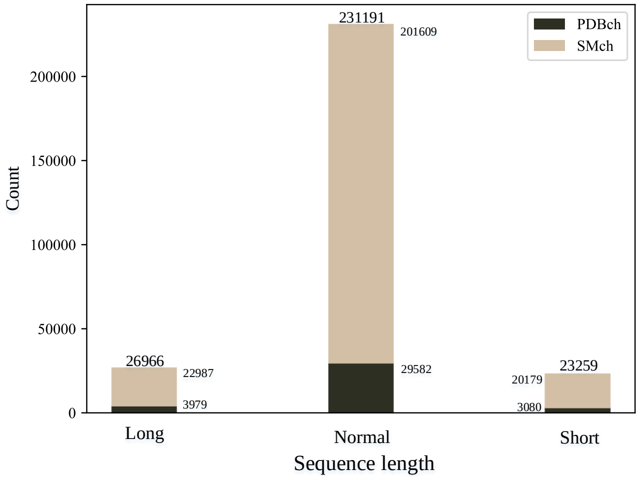

Figure 1: The number of long, normal, and short protein sequence lengths within the PDBch and SMch datasets.

applying the activation function to the scores, an adaptive mechanism is utilized in Algorithm 2 line 13-15, to address the distinctive protein features with varying sequence lengths. Since $\mathbf{h}_v^{(3)}(\mathrm{M})$, $\mathbf{h}_v^{(3)}(\alpha)$, $\mathbf{h}_v^{(3)}(\beta)$ from DH-GNN are obtained with different attention patterns for proteins of varying sequence lengths. Consequently, the attention scores are adaptively selected using a learnable factor ζ. The introduction of ζ is to seamlessly integrate DH-GNN with adaSAB, leveraging adaSAB as a feature enhancement mechanism to harmonize the diverse features from the Main, Extractor$_\alpha$, and Extractor$_\beta$ branches. By introducing ζ, we refine feature selection, allowing Extractor$_\alpha$ and Extractor$_\beta$ to prioritize the most relevant features for their specific tasks (long/short sequence proteins), while still incorporating critical insights from the Main branch.

B

According to the provided information, a protein sequence containing more than 500 amino acids (AAs) is considered a lengthy sequence, while a sequence comprising 100 to 500 AAs is defined as a normal sequence, and a sequence with fewer than 100 AAs is classified as a short sequence. Fig. 1 elucidates the proportional distribution of these different protein sequence lengths in the PDBch and SMch datasets, where 9.5% are lengthy sequences, 8.2% are short sequences, and the remaining sequences fall into the normal category.

C

Results of BP, MF, CC under 40% and 70% ratios with Fmax, AUPR, Sim are shown in Fig. 2.

Table 1: Model Performance comparison of BP, MF, and CC on AUPR on AFch test set.

Model	AUPR		
	BP	MF	CC
DeepGO	20.0	45.8	24.1
DeepFRI	12.6	34.2	17.5
HEAL	19.6	48.8	**28.3**
Struct2Go	<u>21.2</u>	**50.0**	28.1
MEGA-GO	**22.7**	<u>51.4</u>	<u>27.6</u>

The best and second-best results are in bold and underlined(% is omitted in the result.)

D

By initially screening for proteins with less than 30% sequence similarity, we effectively eliminate those that are highly homologous, thus reducing potential redundancy in our analysis. Following this, applying a less than 50% structure similarity threshold for clustering is a strategic choice to ensure a certain degree of distinct structure among the selected proteins.

Within all the results in Fig. 3, the evaluation metrics rank on MEGA-GO are competitive compared with other methods. However, the rates of Fmax, AUPR, and Smin of all the methods are reduced by using sequence similarity below 30% and structure identity below 50%. This is because the protein features tend to be distinct, some proteins that share important functional or structural features may be excluded. As a result, the performance leads to decay, where the model encounters a more heterogeneous set of proteins, making it challenging to identify common patterns or relationships. The distinct nature of these proteins means that the model cannot effectively generalize from the training data, as each protein appears to be an independent entity rather than part of a cohesive group with shared traits. Therefore, we chose 50% instead of continuing to choose a lower structure identity threshold for the experiment.

E

Results in Tab. 1 are model trained on the SMch and PDBch datasets and tested on the AFch test set.

F

The core concept of MEGA-GO focuses on effectively handling both long and short protein sequences. However, these sequences represent only 9.5% and 8.2% of the dataset, respectively (as detailed in section B). This imbalance can lead to biased model performance, as models may favor the more abundant categories. To address this issue, we implement a strategy that involves

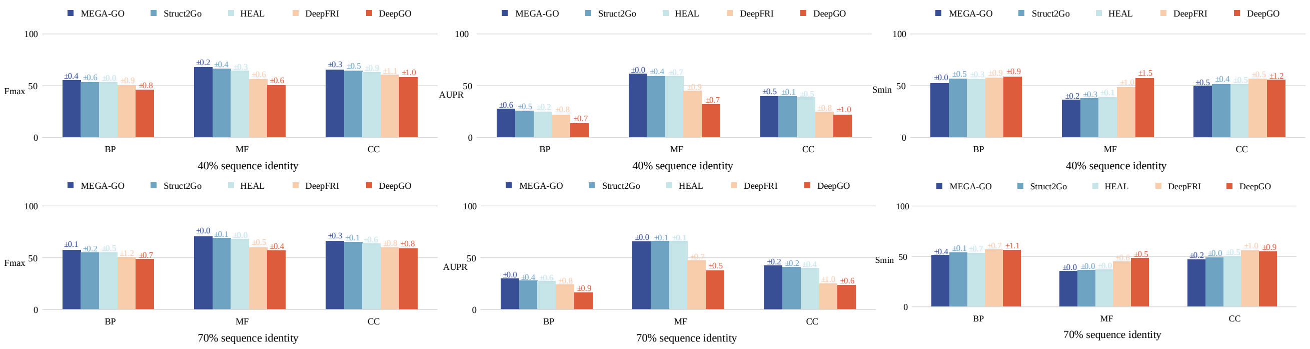

Figure 2: Generalization ability comparison of BP, MF, and CC on Fmax, AUPR, and Smin using 40% and 70% sequence identity threshold, under the train-validation-test split ratio of 8:1:1.

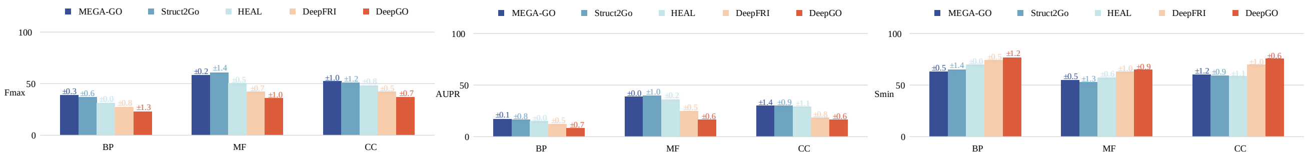

Figure 3: Generalization ability comparison of BP, MF, and CC on Fmax, AUPR, and Smin using lower 30% sequence identity threshold then conduct lower 50% structure identity threshold, under the train-validation-test split ratio of 8:1:1.

Table 2: Cross-validation Experiment

Normal Sequence Protein Data Chunk	AUPR		
	BP	MF	CC
1	33.0	68.8	45.1
2	32.9	68.1	44.7
3	34.2	69.6	45.0
4	32.7	67.8	43.3
5	32.8	68.0	44.9
6	33.9	69.5	44.4
7	33.5	69.2	44.6
8	34.1	69.8	44.8
Avg.	33.4	68.9	44.6

"Chunk" stands for chunking the normal sequence protein data into eight folds, resulting in the approximately ratio of long, normal, short to be 1:1:1; "Avg." stands for the average rate of eight data chunks (% is omitted in the result.)

Table 3: Temporal hold-out evaluation

Model	AUPR (2019)			AUPR (2020)			AUPR (2021)		
	BP	MF	CC	BP	MF	CC	BP	MF	CC
HEAL	29.8	66.7	41.8	<u>27.3</u>	62.8	38.5	26.6	60.4	38.1
Struct2Go	<u>30.1</u>	<u>68.2</u>	<u>42.5</u>	27.0	<u>65.6</u>	<u>40.1</u>	<u>27.0</u>	**63.6**	**38.9**
MEGA-GO	**33.4**	**68.9**	**44.6**	**29.5**	**65.7**	**42.0**	**29.0**	<u>61.2</u>	<u>38.6</u>

The best and second-best results are in bold and underlined (% is omitted in the result.)

random sampling to create a more balanced dataset with an approximately equal ratio of long, normal, and short protein sequences (1:1:1).

To further mitigate potential bias in the evaluation due to the random allocation of samples into training and test sets, we adopt a robust form of cross-validation. Specifically, the normal sequence dataset is divided into eight non-overlapping subsets. This chunking allows us to systematically evaluate the model's performance while ensuring that each subset serves as a unique test set. The final performance metric of MEGA-GO is calculated as the average of the results obtained from these eight different training and testing iterations. This method not only helps to reduce variance in the performance estimates but also provides a more reliable assessment of the model's ability to generalize across different protein sequence lengths and types. The results of this evaluation are summarized in Tab. 2, illustrating the effectiveness of the MEGA-GO model in handling protein sequences of varying lengths.

G

Tab. 3 presents the temporal hold-out evaluation comparing MEGA-GO with the existing SOTA methods: HEAL and Struct2Go. These three methods exhibit a certain degree of performance decline across different years. Specifically, the accuracy of each method decreases when tested on protein annotations from 2020 and 2021 compared to the 2019 annotations. This trend underscores the challenges faced in adapting to evolving protein annotation landscapes over time.

H

Fig. 4(a) illustrates the impact of including adaAF on AUPR scores, highlighting that optimal performance is

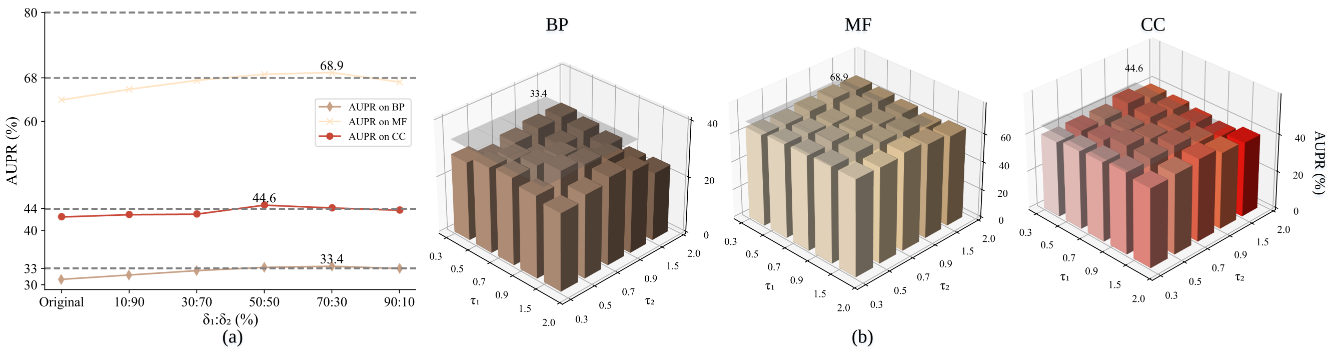

Figure 4: (a). Various ratios of δ_1:δ_2 reach the optimal AUPR score on BP, MF, and CC, with different values of τ_1 and τ_2. "Original" denoted as adaAF is excluded from MEGA-GO, by solely fusing the two feature embeddings $V_{\text{One-hot}}$ and $V_{\text{ESM-1b}}$ together in Eq. (3). (b). The highest AUPR scores on the BP, MF, and CC tasks are 33.4%, 68.9%, and 44.6% respectively, using $\tau_1 = 0.7$, $\tau_2 = 0.5$, under a set of ratios of δ_1:δ_2 = 70%:30% on BP, using $\tau_1 = 0.7$, $\tau_2 = 0.5$, under a set of ratios of δ_1:δ_2 = 50%:50% on MF, $\tau_1 = 0.5$, $\tau_2 = 0.5$, under a set of ratios of δ_1:δ_2 = 70%:30% on CC.

achieved with a balanced feature ratio between $V_{\text{One-hot}}$ and $V_{\text{ESM-1b}}$. Fig. 4(b) presents AUPR scores for different values of τ_1 and τ_2, showing consistent results across selected thresholds while demonstrating the model's focus on long and short protein sequences under certain noise conditions.
